# Dynamic circulating tumor DNA quantificaton for the individualization of non-small-cell lung cancer patients treatment

**DOI:** 10.18632/oncotarget.20016

**Published:** 2017-08-07

**Authors:** Mariano Provencio, María Torrente, Virgina Calvo, Lourdes Gutiérrez, David Pérez-Callejo, Clara Pérez-Barrios, Miguel Barquín, Ana Royuela, Begoña Rodriguez-Alfonso, Miguel Sotelo, Juan Luis Cruz-Bermúdez, Miriam Mendez, Alberto Cruz-Bermúdez, Atocha Romero

**Affiliations:** ^1^ Medical Oncology Department, Hospital Universitario Puerta de Hierro-Majadahonda, Majadahonda, Spain; ^2^ Molecular Oncology Laboratory, Biomedical Sciences Research Institute, Hospital Universitario Puerta de Hierro-Majadahonda, Majadahonda, Spain; ^3^ Biostatistics Department, Biomedical Sciences Research Institute, Hospital Universitario Puerta de Hierro-Majadahonda, Majadahonda, Spain; ^4^ Nuclear Medicine Department, Hospital Universitario Puerta de Hierro-Majadahonda, Majadahonda, Spain; ^5^ Medical Oncology Department, Hospital Infanta Cristina, Parla, Spain; ^6^ Information Technologies Department, Hospital Universidad Politécnica de Madrid, Madrid, Spain

**Keywords:** cfDNA, TKI, personalized medicine, lung cancer, liquid biopsy

## Abstract

**Background:**

Liquid biopsy has evolved from being a promising line to becoming a validated approach for biomarker testing. However, its utility for individualization of therapy has been scarcely reported. In this study, we show how monitoring levels of EGFR mutation in plasma can be useful for the individualization of treatment.

**Results:**

Longitudinal EGFR mutation levels in plasma always correlated with tumor response ascertained by RECIST criteria. Moreover, decreasing EGFR mutation levels were detected in all patients benefiting from locoregional radiotherapy, whereas the opposite occurred when a patient progressed soon after radiotherapy treatment. Similarly, increasing EGFR mutation levels anticipated disease progression after TKI dose reduction, discontinuation of treatment, or reduced bioavailability due to drug interactions. In addition, EGFR mutation levels were useful to monitor treatment outcome of new therapies and constituted a decisive factor when the clinical situation of the patient did not correlate with responses ascertained by radiologist. Finally, our results indicate that cancer associated body fluids (pleural, pericardial or cerebrospinal fluid) are certainly a suitable source for biomarker testing that can extend EGFR mutation detection to biofluids other than blood.

**Materials and Methods:**

A total of 180 serial plasma samples from 18 non-small-cell lung cancer patients who carried an activating EGFR mutation were investigated by digital PCR.

**Conclusions:**

Monitoring levels of EGFR mutation in plasma allows resolving doubts that frequently arise in daily clinical practice and constitutes a major step towards achieving personalized medicine.

## INTRODUCTION

Tyrosine kinase inhibitors (TKIs) targeting the epidermal growth factor receptor (EGFR) have substantially improved the quality of life and survival of advanced non-small-cell lung cancer (NSCLC) patients [1–5]. However, in daily clinical practice, many questions concerning the management of patients undergoing TKI therapies remain unanswered. For example, conventional monitoring of TKI-treated patients relies on morphologic tumor changes, which are generally assessed by computed tomography (CT) scans and response evaluation criteria in solid tumors (RECIST). However, several studies have indicated that RECIST assessment may have some limitations when measuring tumor response to TKIs [6–8]. Oncologists usually continue to treat NSCLC patients harbouring sensitizing EGFR mutation with TKIs for extended periods after RECIST progression [9–11] although this may be continuing ineffective treatments. Indeed, it has been reported that the p.T790M resistance mutation can be effectively detected in plasma of advanced lung cancer patients several months prior to clinical progression [12, 13]. In the same way, dose readjustment due to toxicity is performed empirically and whether drug efficacy is compromised by dose reduction is difficult to measure in daily clinical practice.

This study assessed dynamic changes in EGFR mutation quantification using digital PCR (dPCR) methodology in longitudinally collected plasma samples from NSCLC patients with activating EGFR mutations. We evaluated the clinical information that EGFR mutation levels in plasma samples can provide in the context of daily clinical practice, not only in terms of tumor response, but also with respect to pharmacokinetics and drug interactions and individualization of treatment.

## RESULTS

### Study cohort

This study reports daily clinical practice data obtained from 18 NSCLC patients. Routine follow-up examinations were performed by a medical oncologist every 3 weeks for the first 3 months, and every 12 weeks thereafter or as required according to the oncologist's criteria. The pathological characteristics of the study population are summarized in Table 1.

**Table 1:** Clinico-pathological characteristics of the study population

Median age at diagnosis	59 years (40–73)	*N*	%
***Sex***	*Women*	8	44%
	*Men*	10	55%
***Smoking status***	*Former/quit*	10	55%
	*Never*	8	44%
***Histology***	*Adenocarcinoma*	18	100%
***UICC Stage***	*III*	1	5%
	*IV*	17	94%
***EGFR mutation***	*exon 19 deletion*	12	67%
	*exon 20 insertion*	1	5%
	*L858R*	5	28%
***Number of treatment lines monitored***	*1 line*	3	17%
***throughout the study***	*2 lines*	10	55%
	*3 lines*	5	28%

An average of 10 cfDNA samples were analysed per patient. cfDNA from all blood samples was analysed for the amount of sensitizing EGFR mutation. The p.T790M mutation was concomitantly analysed in all cfDNA samples. ctDNA fluctuations measured as mutated copies/ml or as the ratio mutant allele fraction showed similar patterns. According to our data, in positive case samples the ratio of mutant DNA molecules vs total DNA molecules ranged from 0.10% to 18.5% and the number of mutated copies in positive samples ranged from 144 to 328570 copies/ml.

### EGFR mutation levels to monitor non-standard therapies

Overall, changes in EGFR mutation levels correlated with treatment responses observed in radiology assessments. Our results consistently showed that the detection of the p.T790M resistant mutation in blood, as well as an increment in the quantification of the original sensitizing EGFR mutation in serial plasma samples correlated with the assessment of progressive disease (PD) whereas the opposite occurred in responding patients (Supplementary Figure 1). Importantly, a substantial decrease of p.T790M and the sensitizing mutation levels were notice in a patient (03LSM) treated with cetuximab plus afatinib, who maintained in stable disease (according to radiological assessments) 493 days after the initiation of treatment, suggesting that EGFR mutation levels in plasma might be useful to monitor non-standardized treatments (Supplementary Figure 1). Of note, a total of 19 samples were obtained and analysed from patient 03LSM, which implied obtaining an average of one sample every 26 days during the 493 days of follow up of this patient. This close disease monitoring would be unthinkable if performing exclusively imaging tests.

### EGFR mutation levels tracking as a tool to monitor dose reduction and drug interactions

**Figure 1:**
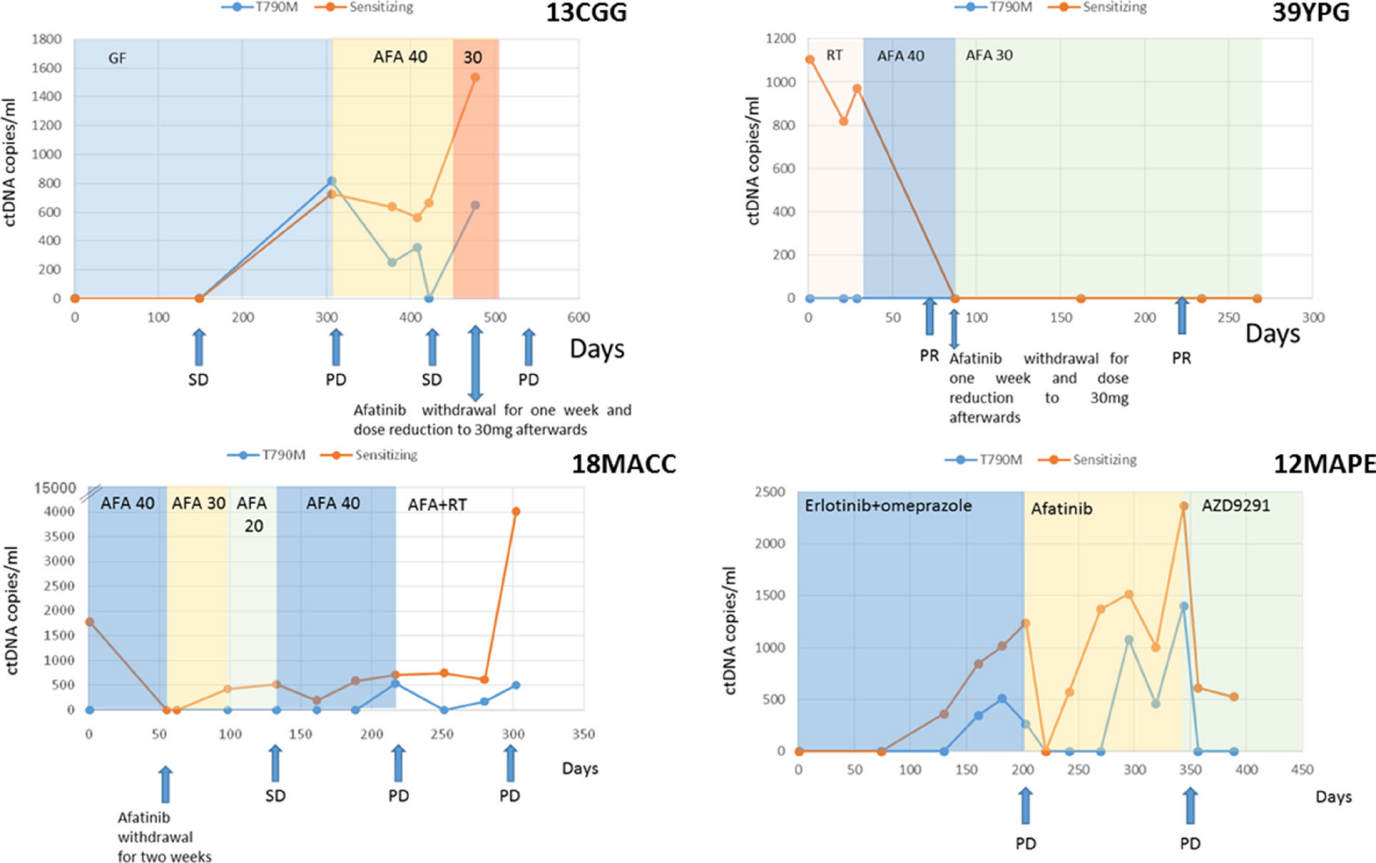
ctDNA levels (copies/ml) of patients 13CGG, 29YPG, 18MACC and 12MAPE Therapies are denoted by different colored shading. Disease status as ascertained on a CT-scan at different time points is marked with arrows. In cases 13CGG and 18MACC an increase in ctDNA is observed after TKI dose reduction correlating with subsequent tumor progression. Increasing levels on ctDNA in case 12MAPE suggest a possible interaction between erlotinib and omeprazole. (Abbreviations: SD = stable disease, PR = partial response, PD = progressive disease, GF = gefitinib, AFA = afatinib, PMT = Pemetrexed, E = erlotinib, RT = radiotherapy).

**Figure 2:**
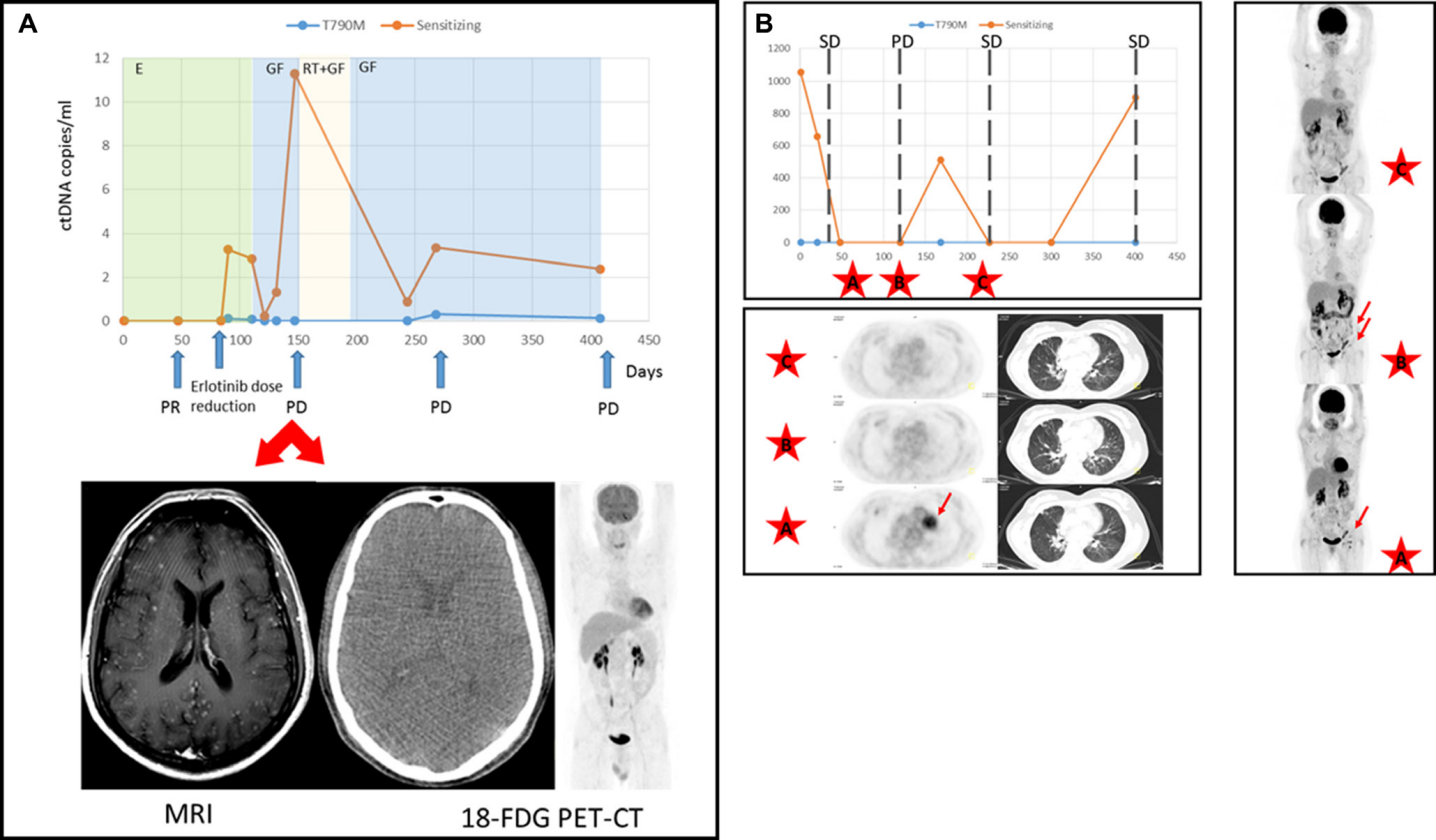
Discrepancies in the assessment of tumor response between ctDNA and TAC (**A**) ctDNA levels (copies/ml) of patient 07AB. Therapies are denoted by different colored shading. Disease status as ascertained on a CT-scan at different time points is marked with arrows. As shown, the 18-FDG PET-CT scan at day 150 failed to detect brain metastases that were however diagnosed by MRI. (Abbreviations: PR = partial response, PD = progressive disease, GF = gefitinib, RT = radiotherapy). (**B**) Mutation tracking profile of case 11MCMS and treatment outcome assessed according to RECIST criteria v.1.1. SD = stable disease, PD = progressive disease. 18-FDG PET-CT scans at time points are marked with a discontinuous line. Images from data points A, B and C are presented. Lung tumor and adenopathies are denoted by arrows.

Dose adjustments were required in 5 patients (04BAA, 07AB, 18MACC, 13CGG and 39YPG). In four of them (04BAA, 07AB, 07MACC and 13CGG), increased EGFR mutation levels were detected after TKI dose readjustment (Figures 1 and 2), and correlated with a subsequent diagnosis of PD. In contrast, no sensitizing mutation increase was observed in the remaining patient (39YPG) after TKI dose reduction and the patient remained in partial response (PR) (Figure 1). In addition, in three cases (04BAA, 07AB and 07MACC) a drop in EGFR mutation levels were observed following the reintroduction of TKI at full dose.

Finally, one case (12MAPE) corresponded to a patient receiving erlotinib who was concomitantly taking omeprazole without medical indication, and increasing levels of EGFR mutation were detected during co-administration of both drugs. As shown in Figure 1, a reduction to undetectable levels in EGFR mutation levels were demonstrated immediately after omeprazole withdrawal although these levels increased again soon after.

### EGFR mutation levels for monitoring oligometastatic disease treatment

During the study, eight patients (02RGB, 07AB, 17VVM, 05JBP, 18MACC, 19MLCG, 30MMP and 32LICV) received loco-regional radiotherapy (RT). As shown in Supplementary Figure 1, changes in EGFR mutation levels were observed during RT, which correlated with responses ascertained by the radiologist. Importantly, EGFR mutation levels in plasma dropped after RT in all patients (Table 2) except for one case (18MACC) who progressed after 64 days. The short time to progression in the latter case suggests a systemic rather than oligometastatic disease. This could potentially be suspected from the increasing EGFR mutation levels in plasma after RT. Note that in patient 07AB, RT was given with palliative intent, due to the patient´s diagnosis of leptomeningeal carcinomatosis.

**Table 2:** ctDNA fluctuations in patients undergoing radiotherapy (RT) and time to progression after RT (days)

Patient	Progression after RT	Time to progression (days)	ctDNA fluctuation
*02RGB*	no	-	↓
*07AB*	yes	98	↓
*17VVM*	no	-	↓
*05JBP*	yes	223	↓
*18MACC*	yes	64	↑
*19MLCG*	no	-	↓
*30MMP*	no	-	↓
*32LICV*	no		↓

### EGFR mutation levels in patients with doubtful diagnoses

Discrepancies with the imaging studies helped to resolve a doubtful diagnosis in patient 07AB, in which an 18FDG-PET/CT scan failed to detect tumor progression in the brain after second-line treatment with full-dose gefitinib. However, a head MRI confirmed multiple brain metastases which correlated with increased plasma levels of EGFR mutation levels within the same time frame (Figure 2A). Similarly, in patient 11MCMS, 18FDG-PET/CT assessments showed a decrease in size and metabolic activity of the pulmonary nodule that coincided with EGFR mutation quantification. Nevertheless, axillary, inguinal and retroperitoneal lymphadenopathies increased in size and number, and progressive disease was diagnosed after radiological imaging (Figure 2B). Remarkably, pathological evaluation of the inguinal adenopathy revealed an absence of malignancy. Notably, this patient was diagnosed with Sjogren's syndrome and essential mixed cryoglobulinemia.

### Body fluids to improve diagnostic yield of ctdna

In four cases, paired samples were collected from body fluid and plasma. Specifically the cytological evaluation of the pleural and pericardial effusions reveal the presence of lung adenocarcinoma cells in all three cases. Similarly, according to pathologist report the presence of lung adenocarcinoma metastasis in cerebrospinal fluid was confirmed. As shown in Table 3, EGFR mutation levels was always higher in the body fluids compared to plasma. In one case (24MAG) (Supplementary Figure 1) p.T790M mutation was not detected in re-biopsy FFPE or in the corresponding plasma sample. However, p.T790M mutation was detected in the ctDNA isolated from malignant pleural effusion at progression and sensitizing levels dramatically went down after treatment initiation with osimertinib.

**Table 3:** ctDNA mutant allele fraction (%) and ctDNA copies/ml in paired samples from plasma and body fluids

Patient	cthNA Source	p.T790M mutant allele fraction (%)	p.T790M copies/ml	Sensitizing mutant allele fraction (%)	Sensitizing copies/ml
***16JLPG***	Plasma	ND	ND	0.345	1804.5
	CSF	ND	ND	6.431	785.25
***27FJAR***	Plasma	0.144	436.812	0.524	676.062
	PE	10.253	334355.500	20.74	510110
***24 MAG***	Plasma	ND	ND	0.427	409.625
	PE	3.18	62225.17	9.66	23080.38
***35MJSS***	Plasma	0.11	155.56	0.80	1259.87
	PCE	9.66	3360850.33	26.88	9607335.10

Abbreviations: ND = not detected, CSF = cerebrospinal fluid, PE = pleural effusion, PEC pericardial fluid).

## DISCUSSION

The majority of previous liquid biopsy studies in patients with EGFR mutations have primarily reported the feasibility of detecting resistance and sensitizing EGFR mutations and how much earlier tumor progression can be predicted using liquid biopsy than CT-scans [12–16]. However, the usefulness of EGFR mutation detection in plasma may not be limited to the early detection of progression, as it could also assist clinicians in daily clinical practice in terms of dose readjustments and drug interactions, among other aspects. For example, where there are no differences in the clinical effectiveness of different TKIs, in most cases the choice relies either on the oncologist's experience and personal decision, or the expected toxicity. In this respect, we know that although the frequency of all-cause severe adverse effects is somewhat similar among TKIs, they may have differing toxicity profiles [17, 18]. Until now, whether dose reductions after adverse events could influence patient survival was unknown, since these events are relatively rare (less than 6%). From our data, it can be speculated that EGFR mutation level quantification can, at least partially, assist clinicians to lower treatment dose minimizing undesired outcomes. As presented, increasing EGFR mutation levels anticipated disease progression after TKI dose reduction whereas EGFR mutation levels dropped when patient maintained response despite dose reduction. Similarly, we report the clinical utility of EGFR mutation levels in plasma to detect drug-drug interactions that we know might occur even with some common, commercially available, soft drinks [19, 20]. As shown in Figure 1, increasing plasma levels of EGFR mutation were found in a patient (12MAPE) who was concomitantly taking erlotinib and omeprazole, suggesting that omeprazole may reduce effectivity of erlotinib upon co-administration.

We similarly consider that EGFR mutation levels in plasma could help to better redefine the use of determined treatment combinations, as occurred with patient 03LSM, who was treated with afatinib plus cetuximab after progression to afatinib. The rationale of this combination is based on solid preclinical data indicating that concomitant administration of the two drugs can eventually overcome p.T790M-mediated resistance [21]. However, in a phase Ib trial, dual inhibition of EGFR with afatinib and cetuximab was modestly active among patients with acquired resistance with an objective response rate of 29% and median progression free survival of 4.7 months (95% confidence interval, 4.3–6.4) [22]. Consequently, this combination has not been extended to routine clinical practice. Patient 03LSM exemplifies how tumor monitoring through liquid biopsy can open up new perspectives for the use of new therapies, anti-EGFR re-challenge, or even N-of-1 trials [23]. On one hand, liquid biopsy reduces the time for treatment outcome monitoring (from 4 weeks when based in imaging tests results to 5 days when based in liquid biopsy tests results, in our institution). On the other, changes in EGFR mutation levels in plasma can be observed within days (data not shown). This enables a closer real time follow up, getting ahead of the radiological response, and therefore improving decision-making regarding treatment selection, modification or continuation.

Finally, our results indicate that cancer associated body fluids are certainly a suitable source for biomarker testing that can extend EGFR mutation detection to bio-fluids other than blood (Table 3). As proved with patient 24MAG, who presented a p.T790M negative biopsy both in the solid tumor and blood, but was positive in the malignant pleural effusion. Malignant pleural effusions are frequently observed in advanced lung adenocarcinomas [24] and thoracentesis may be necessary for diagnosis and treatment. However, pleural fluid is usually discarded after the removal of cell component for cytological examination despite being helpful for EGFR mutation detection.

In conclusion, sensitizing and resistance mutation quantification in plasma throughout the course of a NSCLC cancer allows us to resolve doubts that frequently arise in daily clinical practice and reduce time for decision, which constitutes a major step towards personalized medicine.

## MATERIALS AND METHODS

### Study population and data management

A total of 18 cases from NSCLC patients were included in the study. Written informed consent was obtained from every patient. Eligible patients were both male and female patients with a pathologically confirmed diagnosis of stage IIIB-IV NSCLC tumor with an EGFR mutation in primary tumor tissue. A complete staging workup was performed prior to recruitment into the study. Blood samples were collected as follows: at diagnosis, when patients returned for re-evaluation and when appointed by a medical oncologist due to uncertainties about a patient's clinical status, radiological assessments, toxicity events or TKI dose reduction. Demographic characteristics, clinico-pathological features, tumor mutation status, vital status, disease status, drug dose adjustments or discontinuation of medication were collected.

The study was approved by the Hospital Puerta de Hierro Ethics Committee (internal code PI/144-14) and was conducted in accordance with the precepts of the Code of Ethics of The World Medical Association (Declaration of Helsinki).

### Laboratory procedures

For EGFR mutation levels quantification, peripheral whole blood was collected from each subject in a 5 ml EDTA tube containing a gel barrier (PPT^™^, BECTON DICKINSON) to separate the plasma from blood cells after centrifugation. All samples were processed as previously described [14, 25]. cfDNA was extracted using a starting volume of 1 ml of plasma with a Maxwell^®^ RSC instrument (Promega), using the Maxwell^®^ RSC ccfDNA Plasma Kit, as specified by the manufacturer and was eluted in 50 μl of the supplied buffer. In addition, biological fluids with malignant abnormal cytology, including malignant pleural (*N* = 2) and pericardial (*N* = 1) effusions and cerebrospinal fluid (*N* = 1) were also analysed for EGFR mutation levels. Biological fluids were centrifuged and the supernatant was used for cfDNA isolation using the same protocol. Germline DNA was obtained from blood leukocytes with a MagNA Pure LC total nucleic acid extraction kit in a MagNA Pure LC instrument (Roche Diagnostics, Penzberg, Germany). cfDNA samples were then analysed by dPCR using Rare Mutation Assays for p.T790M (AHRSROS), p.L858R (AHRSRSV), p.G719A (AHABH29), p.G719C (AH0JEWC), p.G719S (AHZAGP4), p.H773_V774insH (AH5I7PA), p.D770_N771insG (AH7031Q), p.L747_T751 > P (AHFA92K), p.L747_A750 > P (AHS1PY0), p.E746_T751 > A (AHHS6E0), p.E746_A750delELREA (AHLJ0XO), p.L747_T751delLREAT (AHCTDP3) and p.L747_S752delLREATS (AHGJ78R) on a QuantStudio^®^3D Digital PCR System (Applied Biosystems, South San Francisco, CA), as previously described [14]. A wt control DNA was included in every run.

### Tumor response evaluation

Computed tomography (CT) measurements and magnetic resonance imaging (MRI) were obtained as clinically indicated. The clinical response was evaluated according to RECIST criteria v1.1 combined with a blinded medical judgment of benefits from the treatment. Additionally, whole body 18F-fluoro-2-deoxy-D-glucose-positron emission tomography (18FDG-PET)- CT scans were performed as clinically indicated using a Siemens Biograph 6 True Point PET-CT (Siemens). A 350-450MBq 18F-FDG dose was administered 55-65 min prior to image acquisition. Reconstruction was performed using an iterative method and attenuation/scatter correction.

## SUPPLEMENTARY FIGURES


