# Supplementary material for: Dynamic circulating tumor DNA quantificaton for the individualization of non-small-cell lung cancer patients treatment
Source: Oncotarget. 2017 Aug 7;8(36):60291–8. doi: 10.18632/oncotarget.20016 (PMC5601139; doi:10.18632/oncotarget.20016)
Supplement: Supplementary file 1 [file oncotarget-08-60291-s001.pdf]

# Dynamic circulating tumor DNA quantificaton for the individualization of non-small-cell lung cancer patients treatment

## SUPPLEMENTARY MATERIALS

**Supplementary Figure 1: (see following pages).** Serial EGFR sensitizing and resistance mutation levels and treatment outcome, assessed according to RECIST criteria v.1.1. SD = stable disease, PR = partial response, PD = progressive disease of individual cases. Types of treatment (cytotoxic, biological therapy and/or radiotherapy) are shown in coloured shading. Only patients in which ctDNA was detected and a minimum of 4 plasma samples extracted at different time points are presented.

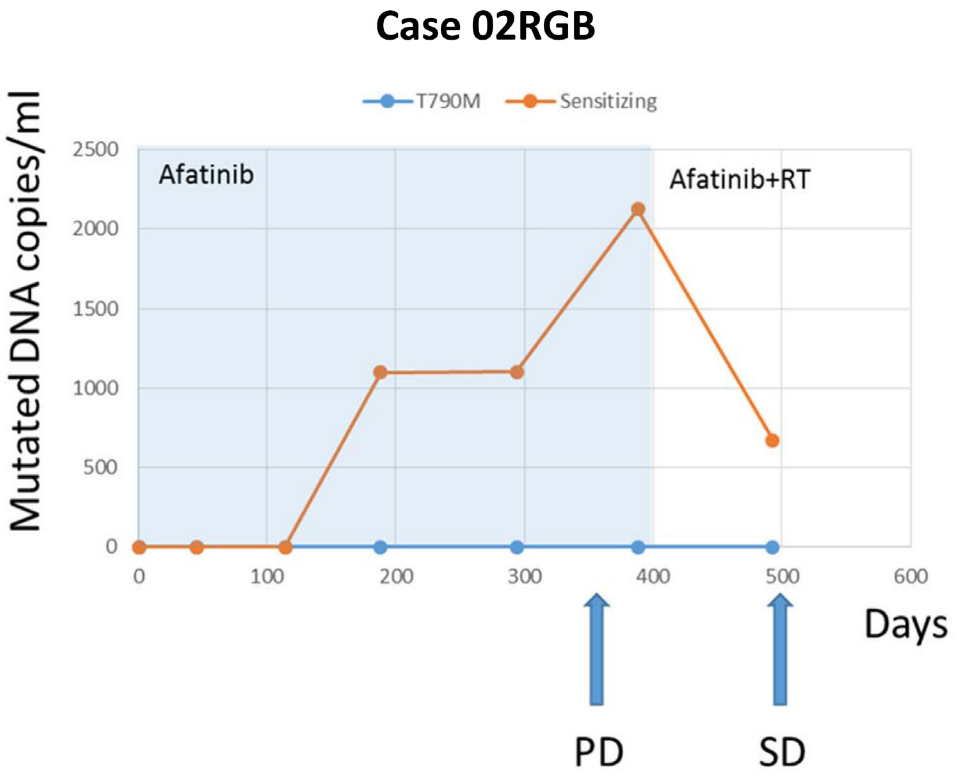


**Supplementary Figure 1-1: 66-year-old female patient diagnosed with lung adenocarcinoma with multiple brain metastases.** Patient started treatment with afatinib 30 mg/day. In March 2015 disease progression was diagnosed at brain level. Her disease established after holocranial radiotherapy (RT).

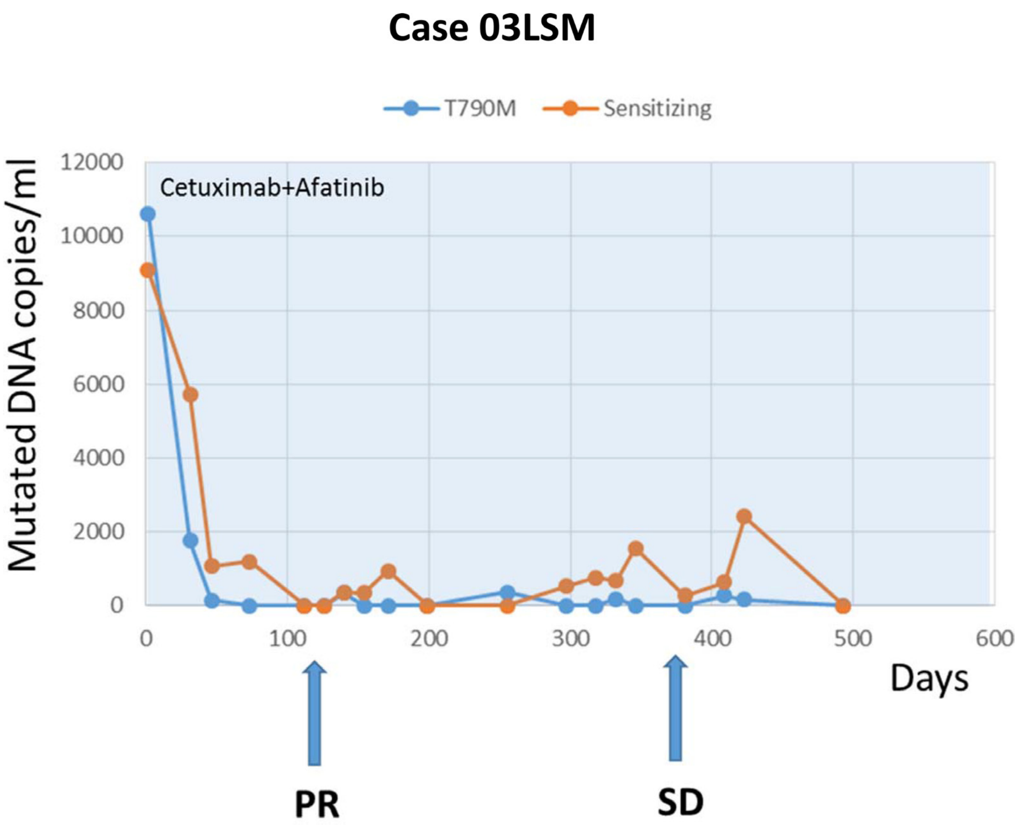


**Supplementary Figure 1-2: Metastatic lung adenocarcinoma 72-year-old male, previously treated with afatinib 40 mg/day.** A CT-scan evidenced a mass in the left hemipelvis. Cetuximab 500 mg/day every 2 weeks was added to treatment initially obtaining a good partial response and a further stabilization of the disease.

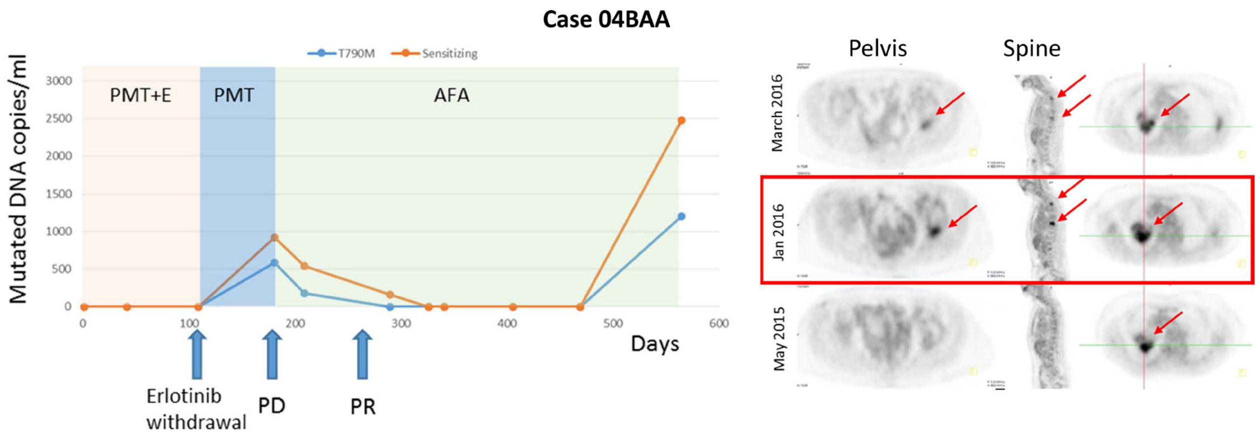


**Supplementary Figure 1-3: 77-year-old female diagnosed with stage IV lung adenocarcinoma, receiving pemetrexed (PMT) plus erlotinib (E) with acceptable tolerance until day 100 when erlotinib (E) was suspended due to severe skin toxicity and asthenia.** Subsequently, pemetrexed (PMT) was maintained alone until disease progression (January 2016-in red). Treatment with afatinib (AFA) was then commenced and a partial response was observed (March 2016). Further tumor progression was detected on day 565. Images obtained in 18FDG-PET/CT scans from May 2015, January (Jan) 2016 and March 2016 show pathological FDG uptake (denoted by arrows) of pelvic and cervical metastases and the lung tumor.

## Case 05JBP

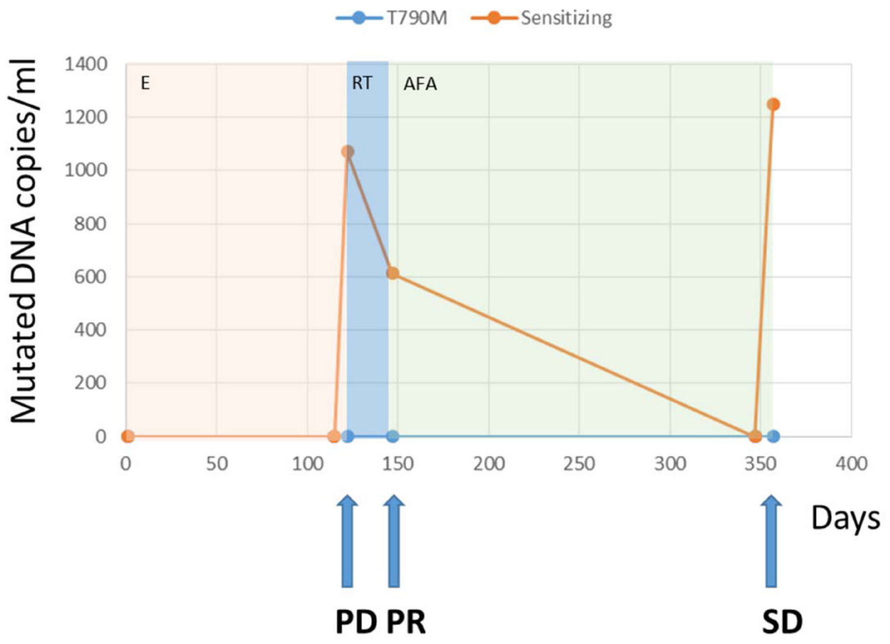


**Supplementary Figure 1-4 : Metastatic lung adenocarcinoma 64-year-old male who started first line treatment with erlotinib (E) 150 mg/day.** A CT scan revealed a pulmonary infiltration and subsequently the patient started esophageal thoracic radiotherapy (RT), extensive to lymph nodes with a dose of 30 Gy. Further imaging scans showed a partial response. Patient initiated afatinib 40 mg/day obtaining disease stabilization. However, an adrenal nodule of new appearance was documented on CT scan on day 350.

## Case 07AB

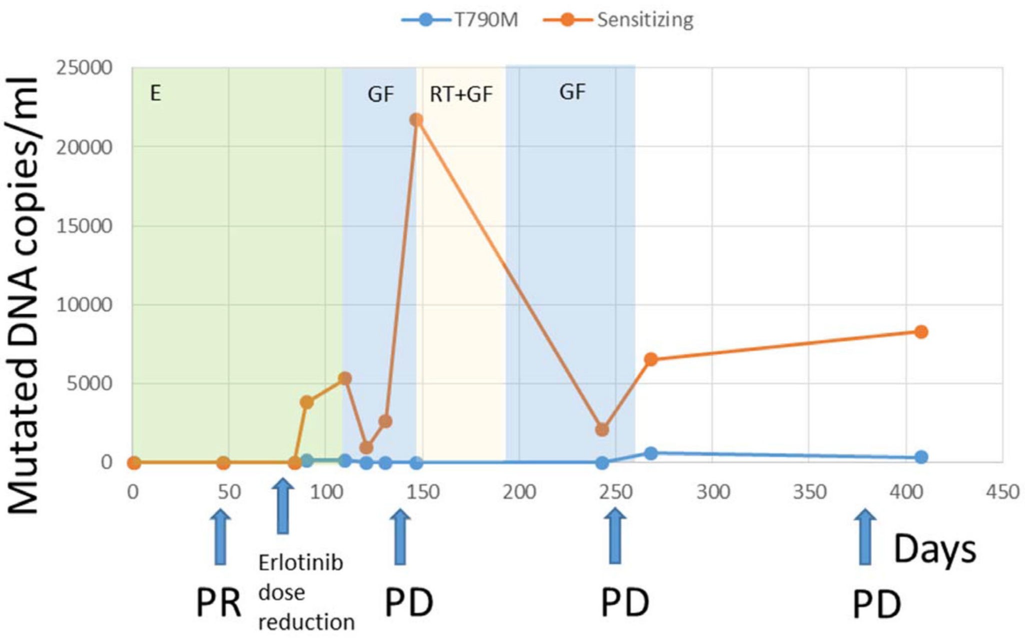


**Supplementary Figure 1-5: Metastatic lung adenocarcinoma 44-year-old male.** Patient started erlotinib (E) 150 mg/day that was later interrupted for two weeks due to acneiform eruption. Subsequently, erlotinib (E) dose was reduced to 50 mg/day but severe skin toxicity was observed and he was finally switched to gefitinib (GF) 250 mg/day. Two weeks later, a brain mass was documented on MRI and patient started receiving radiotherapy (RT). Further disease progression was observed and patient finally died due to leptomeningeal carcinomatosis.

# Case 11MCMS

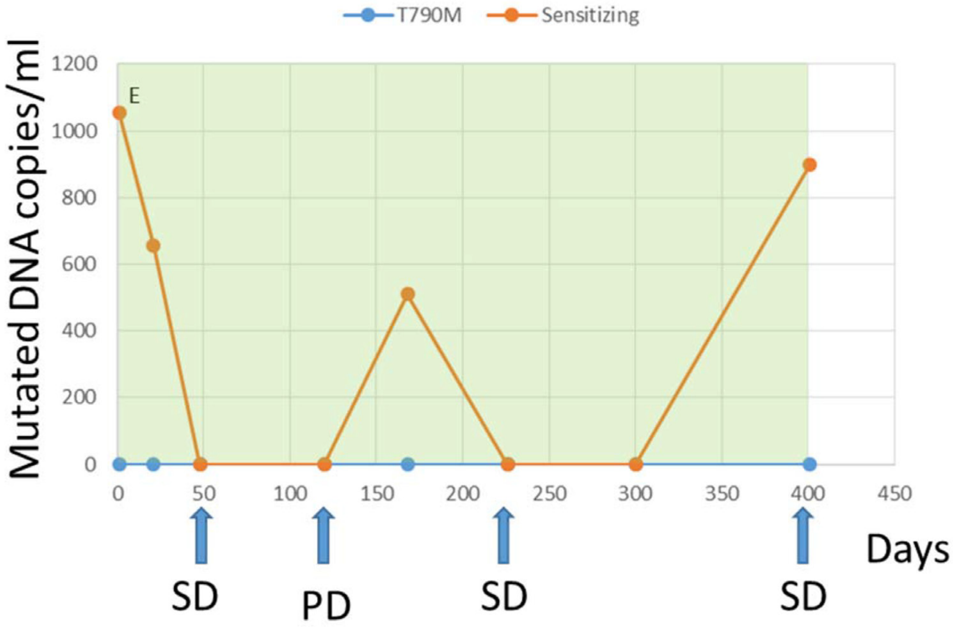


**Supplementary Figure 1-6: Metastatic lung adenocarcinoma 49-year-old female, with previous history of Hodgkin lymphoma treated with radiotherapy.** Patient initiated treatment with erlotinib (E) 150 mg/day with stabilization of the disease. On day 120 an increase in size and activity of several adenopathies was observed on a 18FDG-PET/CT scan. However, anatomopathological evaluation of the inguinal adenopathy revealed absence of malignancy. Of note, this patient was also diagnosed with Sjogren syndrome and essential mixed cryoglobulinemia.

# Case 12MAPE

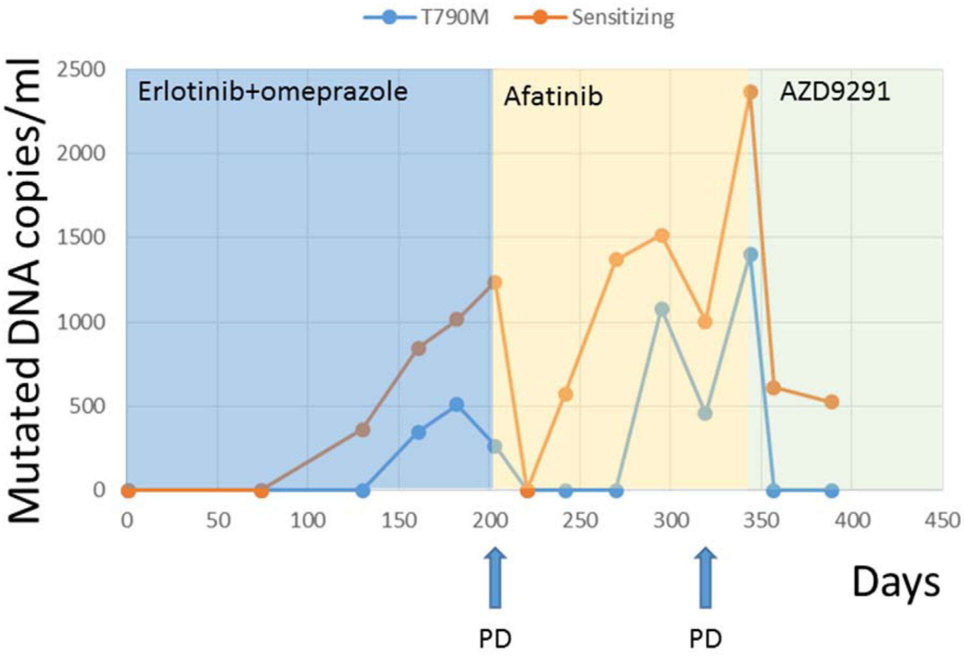


**Supplementary Figure 1-7: Metastatic lung adenocarcinoma 67-year-old male treated with erlotinib (E) 150 mg/day and concomitantly taking omeprazole without medical indication.** As shown, a reduction to undetectable levels in ctDNA was evidenced right after omeprazole withdrawal, although ctDNA levels started to increase soon after and progressive disease was asserted. Patient commenced afatinib and further progression disease was observed. Subsequently, the patient started treatment with AZD9291.

## Case 13CGG

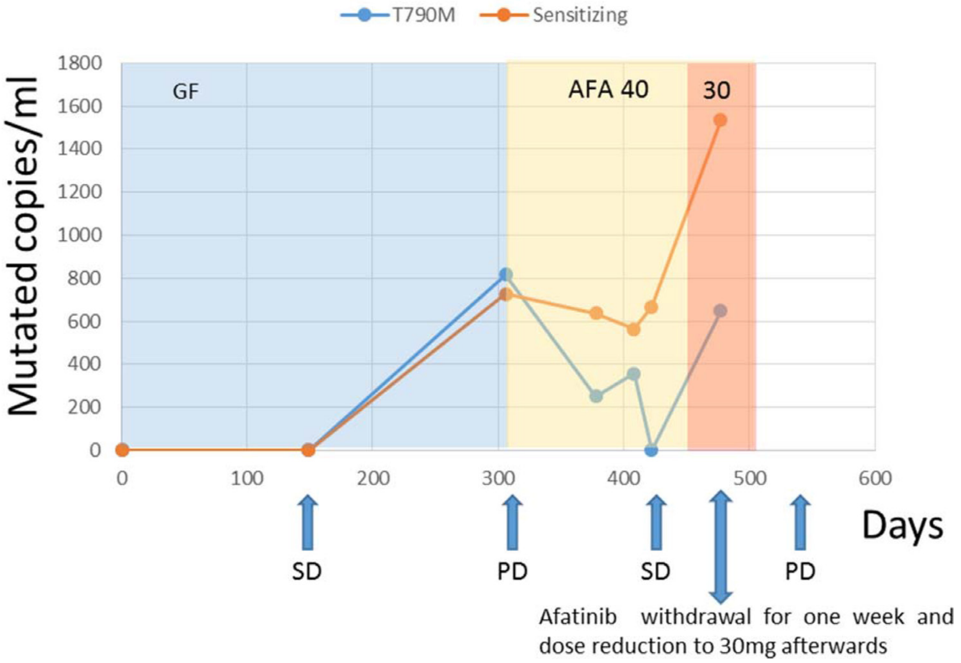


**Supplementary Figure 1-8: Metastatic lung adenocarcinoma 66-year-old female treated with gefitinib (GF) at full dose.** After disease progression treatment was switched to afatinib 40 mg/day but was later stopped for one week due to toxicity which caused afatinib dose reduction to 30 mg/day. Further disease progression was observed.

## Case 16JLPG

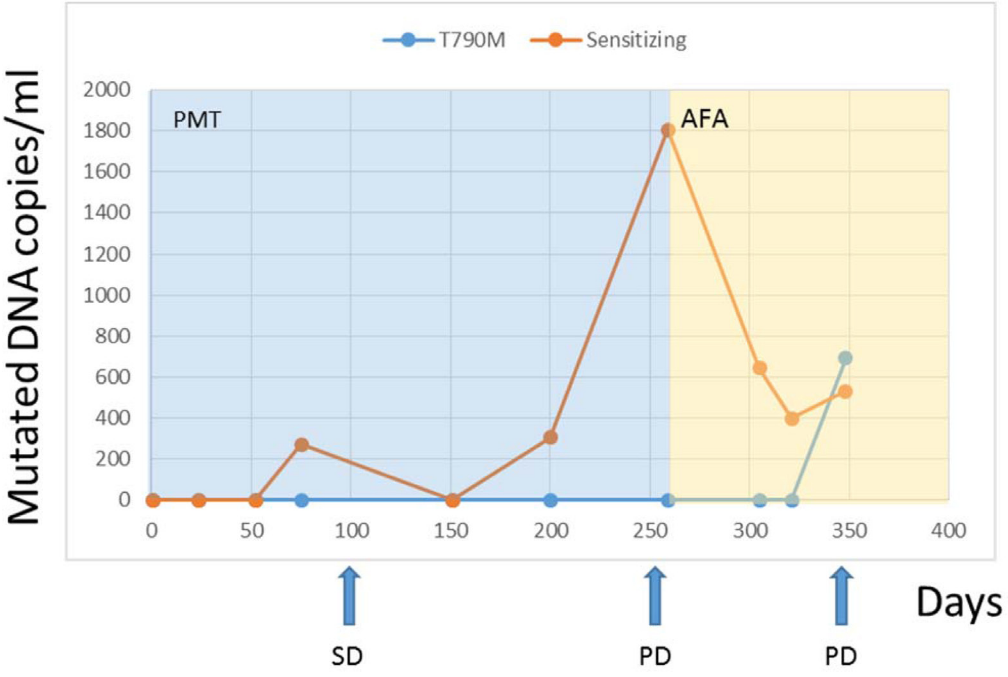


**Supplementary Figure 1-9: 59-year-old male diagnosed with stage IIIB lung adenocarcinoma, with progressive disease confirmed at brain level during treatment with pemetrexed-carboplatin (PMT-C).** Treatment was switched to afatinib 40 mg/day. Further progressive disease was observed on day 349.

## Case 17VVM

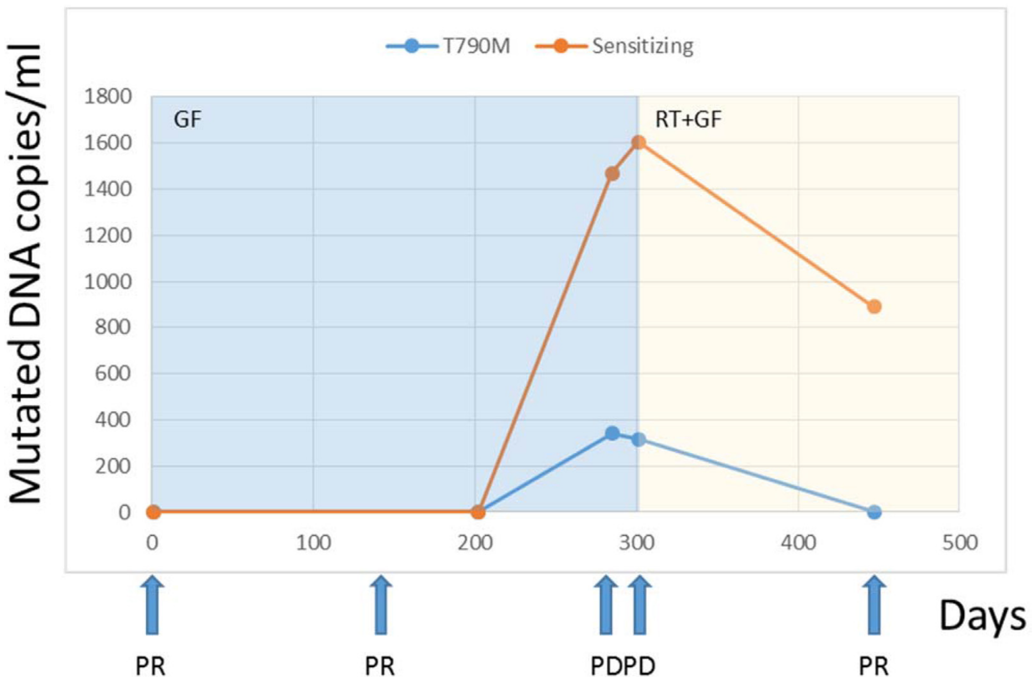


**Supplementary Figure 1-10: 59-year-old women diagnosed with stage IV lung adenocarcinoma and treated with Gefitinib (G) at full dose.** Mild progression was ascertained with CT-scan which revealed an increase in size of the perihilar mass and patient commenced local radiotherapy (RT).

## Case 18MACC

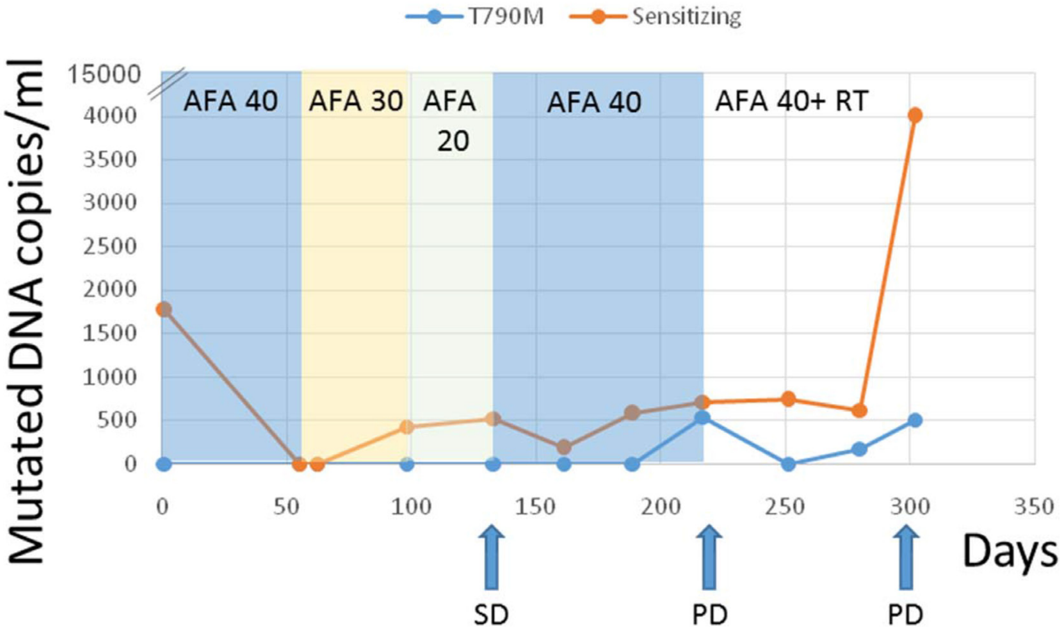


**Supplementary Figure 1-11: Metastatic lung adenocarcinoma 57-year-old male treated with afatinib (AFA) 40 mg/day reached disease stabilization.** Twelve months later, treatment was suspended due to dermal toxicity and recommenced at 30 mg/day dose. A new dose reduction to 20 mg/day was required due to dermal and gastrointestinal toxicity and maintained asthenia. Brain metastases were identified and the patient was treated with local radiotherapy. Finally, leptomeningeal carcinomatosis was diagnosed causing the patient´s death.

# Case 19MLCG

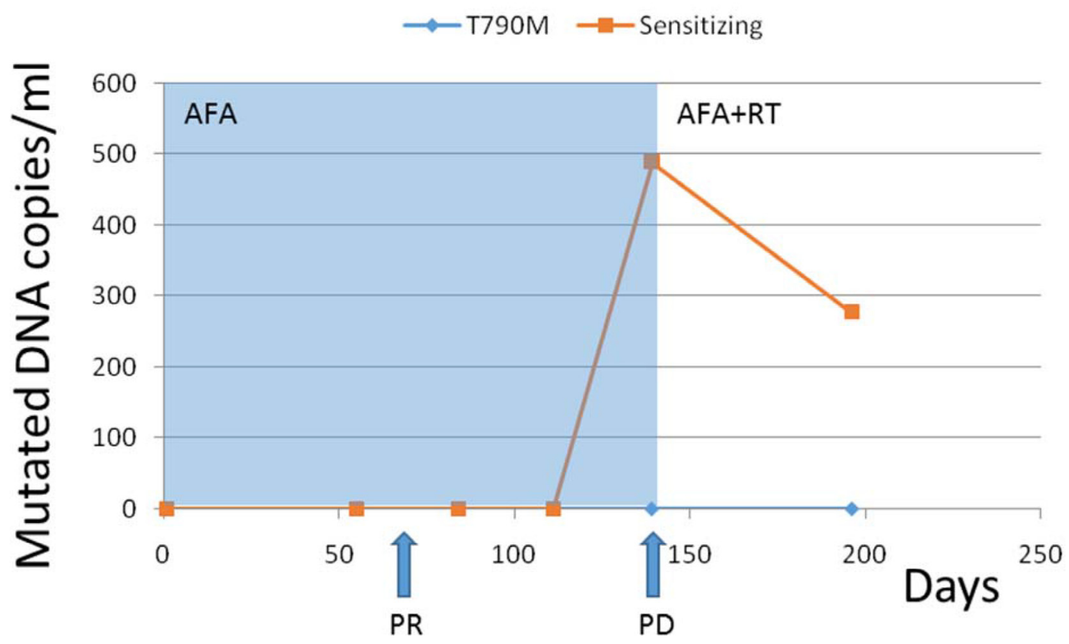


**Supplementary Figure 1-12: 45-year-old female diagnosed with stage IV lung adenocarcinoma and treated with afatinib (AFA) 40 mg/day.** The patient received radiotherapy (RT) after progression to bone metastases.

# Case 24MAG

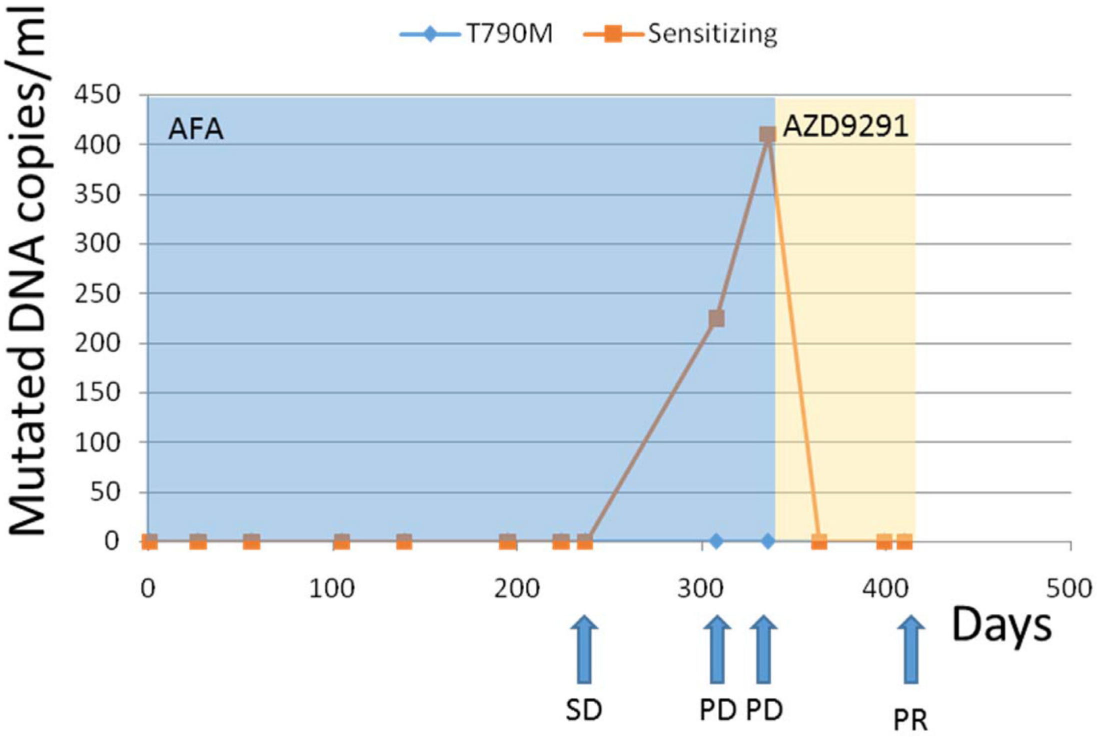


**Supplementary Figure 1-13: 50-year-old male diagnosed with stage IV lung adenocarcinoma, treated with afatinib 40 mg/day until pleural effusion was diagnosed.** Pleural fluid was obtained and analyzed. T790M resistance mutation was not detected in blood but was detected in pleural fluid. Subsequently, treatment was switched to AZD9291.

## Case 27FJAR

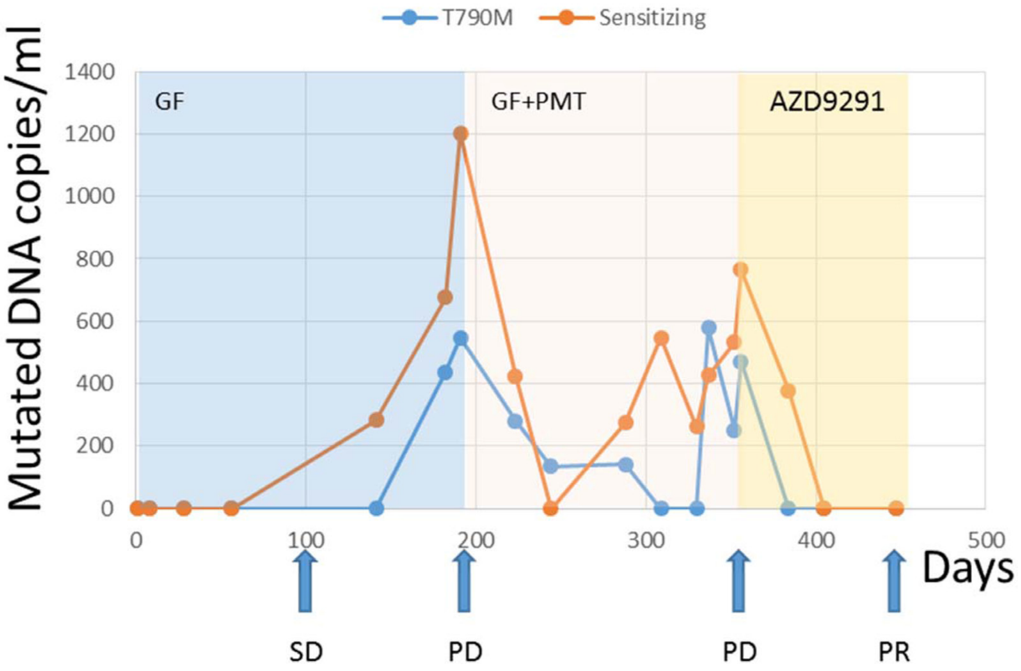


**Supplementary Figure 1-14: Metastatic lung adenocarcinoma 77-year-old male under treatment with gefitinib (GF) 250 mg /day.** Pemetrexed was added to therapy after systemic disease progression. Further progression was documented and patient commenced AZD9291.

## Case 30MMP

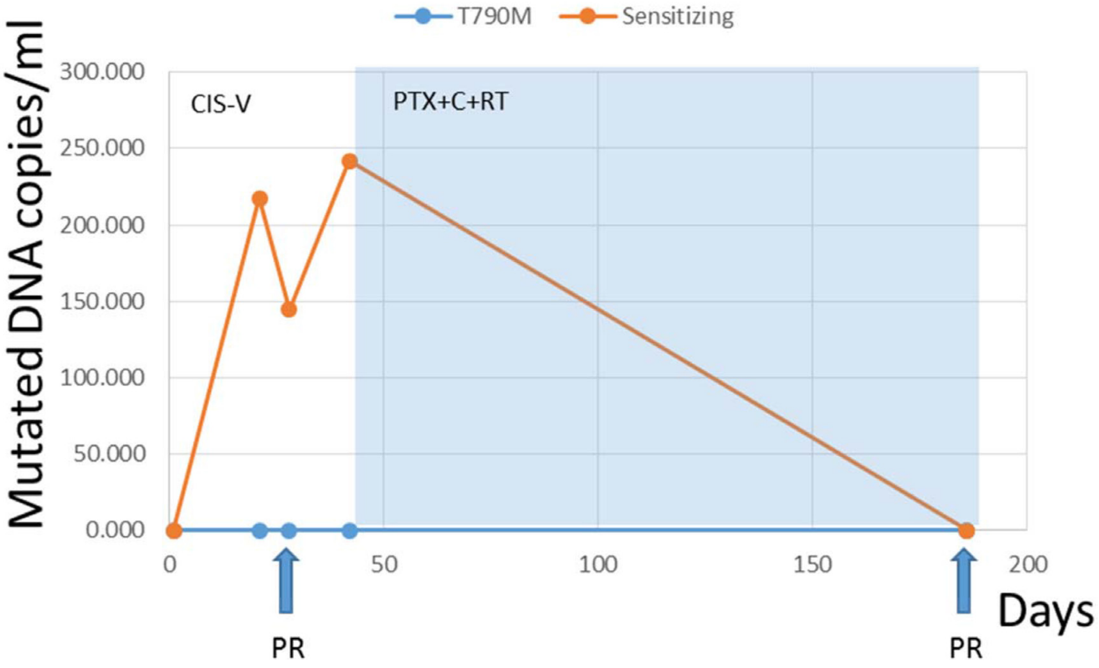


**Supplementary Figure 1-15: 55-year-old male diagnosed with stage IIIB lung adenocarcinoma.** Genotyping of the tumor biopsy revealed the presence of the p.H773_V774insH mutation. Patient initiated chemotherapy scheme with cisplatin-vinorelbine (CIS-V), showing partial response after 4 cycles. Subsequently, the patient received chemo-radiotherapy with paclitaxel (PTX)-carboplatin (C).

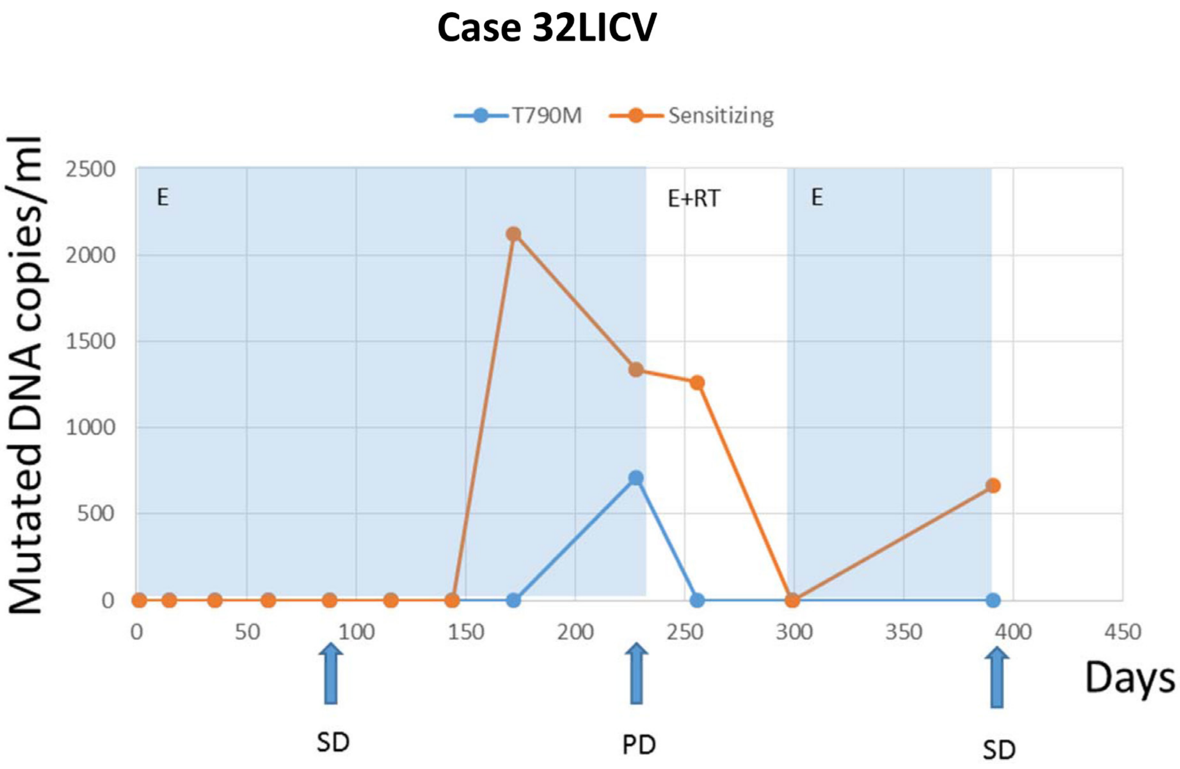


**Supplementary Figure 1-16: 61 year old men diagnosed with stage IV lung adenocarcinoma treated with erlotinib (E).** The patient received local radiotherapy (RT) after mild tumor progression and continued with erlotinib.

# Case 35MJSS

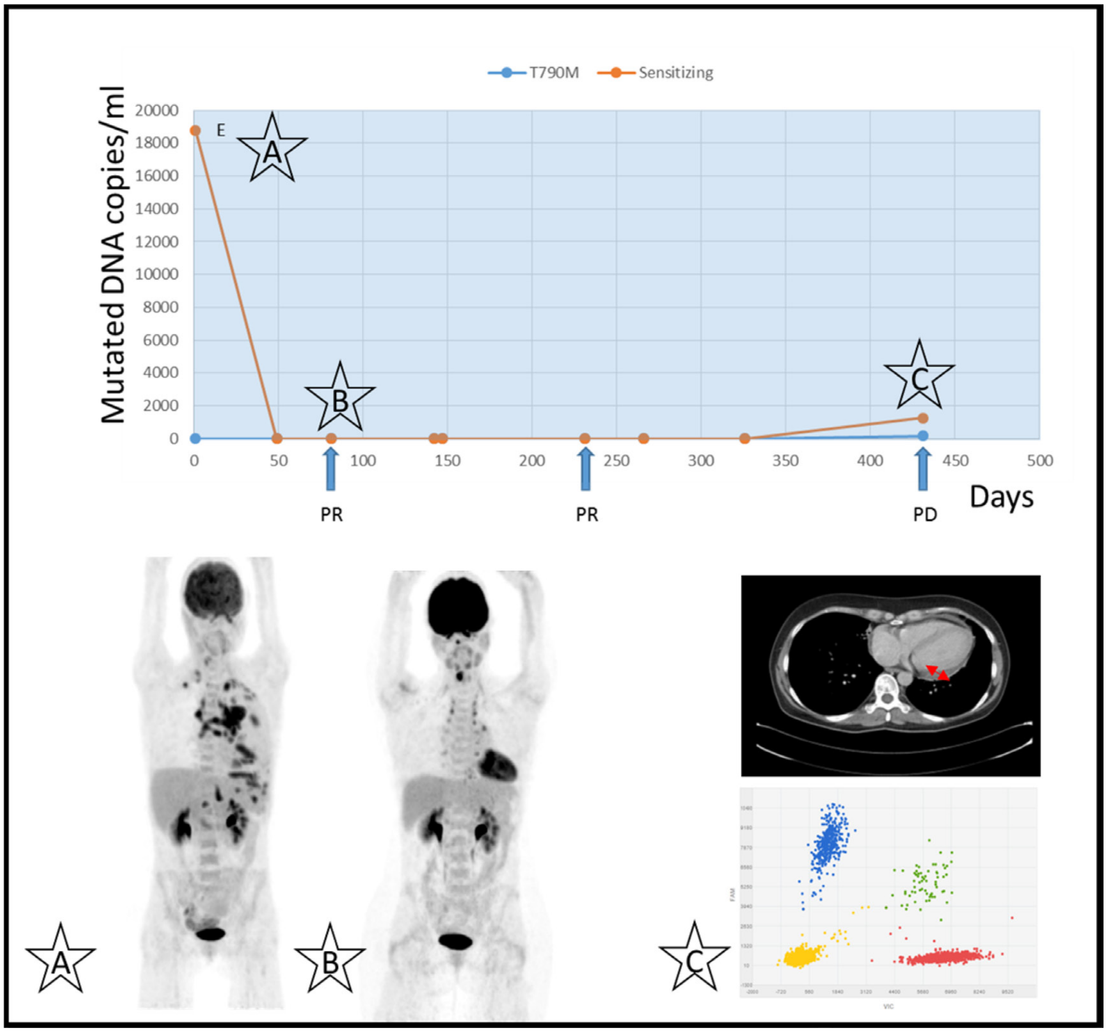


**Supplementary Figure 1-17: Serial measurements of ctDNA (copies/ml) correlating with a positive response observed in scan images.** (A) Basal 18FDG-PET/CT scan showing findings suggestive of malignancy in the lung and pleura, supraclavicular, mediastinal, axillary and retroperitoneal lymph nodes. (B) Second 18-FDG PET-CT scan showing a good response to TKI treatment. (PR = Partial Response). On day 420, the patient attended the emergency department presenting acute abdominal pain. An abdominal X-ray suggested a pericardial effusion that was further confirmed by a CT scan (C) which also showed a discrete increase in size of a hilar adenopathy. Double arrow indicates pericardial effusion seen on CT-scan. The mutation p.T790M was detected in the malignant pericardial effusion as shown in the scatter plot. The p.T790M mutation is labeled with FAM (blue data points), whereas wild-type is labeled with VIC (red data points).

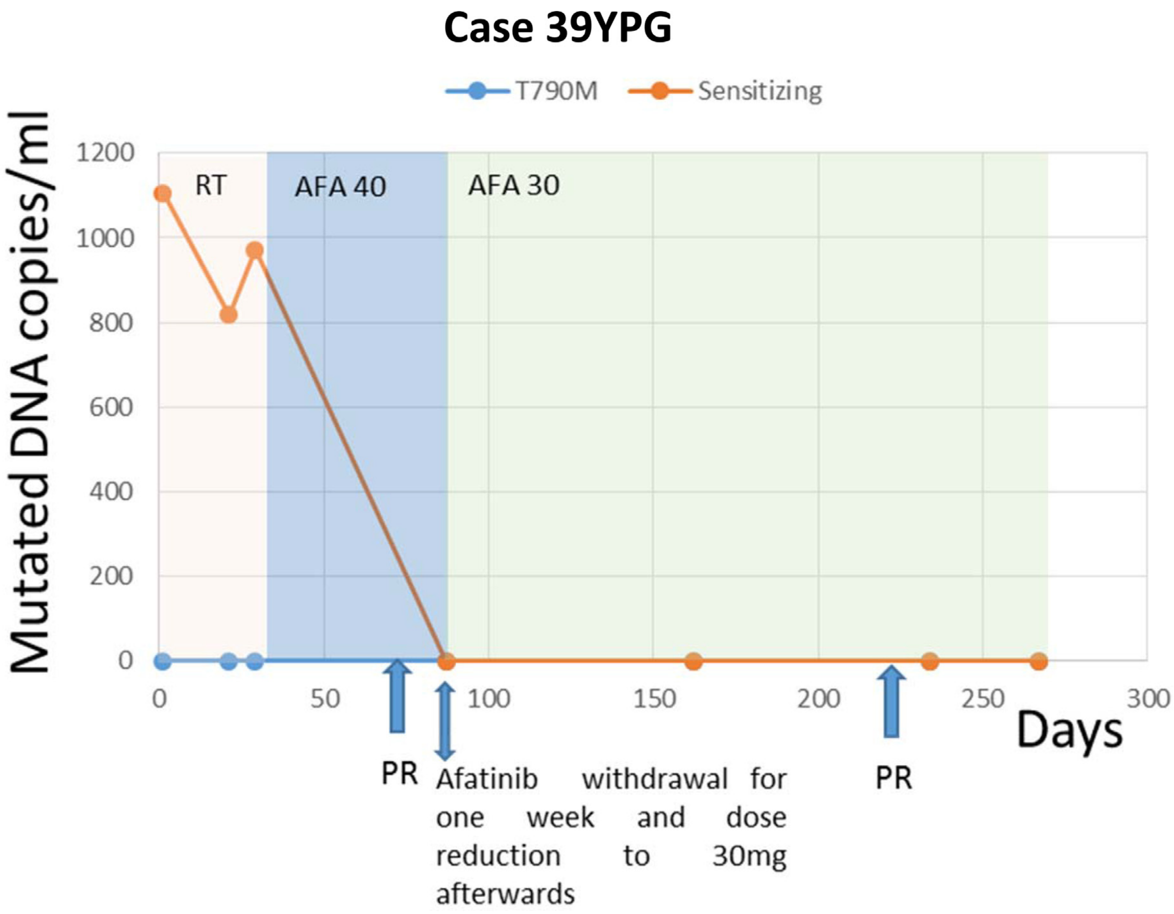


**Supplementary Figure 1-18: 50-year-old female with stage IV lung adenocarcinoma diagnosis.** The patient was first treated with radiosurgery with whole-brain radiotherapy (RT) for brain metastases. Subsequently, she commenced afatinib (AFA) showing a positive response. A dose reduction to 30 mg/day was required due to toxicity.
